# CFO Gender, Corporate Risk-Taking, and Information Disclosure Violations

**DOI:** 10.3389/fpsyg.2022.902472

**Published:** 2022-07-13

**Authors:** Yujie Zhao, Jiaxin Xiong, Jingjing Wang, Nanji Ye

**Affiliations:** ^1^School of Management, Shanghai University, Shanghai, China; ^2^School of Finance, Jiangxi University of Finance and Economics, Nanchang, China

**Keywords:** female CFO, information disclosure violations, CFO power, external monitoring, China, G30, G41, M14

## Abstract

The sex ratio at birth in China exhibits a major occurrence of “missing women” due to the high son preference in Chinese culture. Clearly, the large gender discrepancy in China can be explained not only by ethical, moral, or social fairness theories but also by the economic benefits of women's particular abilities, experiences, and talents. This article examines the influence of female chief financial officers (CFOs) on information disclosure violations in order to highlight women's positive contributions. Our data imply that having a female CFO can dramatically lower the number of companies that fail to disclose information. The results are strong after controlling endogeneity with propensity score matching, Heckman's two-stage self-selection model, and CFO change, as well as controlling the gender of the chairman and chief executive officer, utilizing different study periods, and using exogenous shock. We further examined the moderate effects of CFO power and external monitoring, and we found that CFO power magnifies the negative effect of female CFO on violations; the more the power, the more the negative effect of female CFO on violations. We also found that when the firm has effective external monitoring, there are fewer future infractions of information disclosure.

## Introduction

Working women who are housewives suffer work-life conflicts and a trade-off between work domain and family life domain since traditional Chinese culture prioritizes women's familial obligations as mothers and wives. The pressures from family, community, and country always push women to weigh family against work. As a result, people assume men are better able to focus on work and produce greater results than women, and thus, men are given more resources and opportunities to work. Due to a lack of law and regulation, explicit gender discriminations have been reported in a range of areas in China, including employment and career advancement in the labor market (Kuhn and Shen, 2013; Gao et al., 2016), access to financial resources (Bellucci et al., 2010), education, and personal health (Qi et al., 2016).

Gender discrimination will prevent females from reaching top management positions and reduce women's willingness to work in the short term, while continued discrimination will lead women to be childless or prefer sons over daughters in order to avoid discrimination happening to their children again, both of which will result in an imbalanced population in China in the long run.

To address this issue, the Chinese government has enacted a slew of gender-focused equal opportunity regulations aimed at increasing women's involvement in the workforce. However, the strong effort to reduce gender discrimination is motivated not only by ethical, moral, or regulatory rules but also by the economic benefits of women's particular abilities, experiences, and talents (Zalata et al., 2019). According to studies on business performance/value, having more women on boards improves board independence, monitoring, advising ability, and resources through greater connections of the firms to the external environment (Terjesen et al., 2009). Female executives also have favorable effects on firm performance/value (Liu et al., 2014). Using a unique sample of listed Chinese companies, we investigated whether female chief financial officers (CFOs) help reduce corporate information disclosure violations.

Compared with the female-fraud studies, we focused on CFOs because their duties are financial and they bear the prime responsibility for reporting accurate and timely financial disclosures for the firm (Ham et al., 2017). Many previous studies have found the value of female chief executive officers (CEOs) or Chairman of the Boards in many aspects, such as financing and investing (Huang and Kisgen, 2013; Faccio et al., 2016), accounting policies and regulations (Francis et al., 2015; Zalata et al., 2019), and social responsibilities (Zalata et al., 2019). Despite the fact that the CFO is one of the most significant positions in the company, few studies are conducted on CFOs. This is why we chose to study the effects of female CFOs rather than female CEOs (McGuinness et al., 2017; Grosser and Moon, 2019). We expected CFO characteristics to have a particularly important influence, incremental to CEOs, on financial disclosure, given the CFO's oversight role.

In this article, first we looked at the influence of female CFO on information disclosure violations by using different metrics of violation. We discovered that female CFOs are more cautious with information disclosure than their male counterparts, owing to the fact that companies with female CFOs are less likely to commit information disclosure infractions. Our findings are robust to different measures of information disclosure violations, using the propensity score matching (PSM) method, controlling for the fact that the gender of the CFO is an endogenous choice by using a Heckman self-selection model, using the dissociative identity disorder (DID) test with a sample of firms experiencing a transition from male to female CFOs or vice versa, controlling the gender of the CEO and Chairman of the Board, implementing different samples, and considering an exogenous shock.

Second, we investigated the moderate effect of CFO power on the relationship between CFO gender and disclosure violations. Our results demonstrate that CFO power enhances the female gender's ability to reduce disclosure violations. Therefore, the more powerful a female CFO is, the more crucial their risk-averse personality trait and ethical sensitivity can be in lowering violations, resulting in fewer corporate infractions.

Finally, we investigated the moderate effect of effective external monitoring on the relationship between CFO gender and disclosure violations since effective monitoring can restrain executive opportunities to capture personal benefits. Our results show that external monitoring can amplify the effect of female gender on lowering disclosure violations. As a result, the negative relationship between female CFOs and information disclosure violations is more profound for firms with poorer external monitoring.

Our results contribute to the literature in two ways. First, compared with the current female-fraud studies, we extended the literature focusing on CEO effects on corporate decision-making. Whereas earlier studies have focused on the impact of CEOs or Chairman of the Board of Directors (Palvia et al., 2015), CFOs have more influence and power in corporate financial reporting because they are the direct executors and controllers of the firm's financial disclosure (Jiang et al., 2010). Given the CFO's oversight role, we expected CFO characteristics to have a particularly important influence, incremental to CEOs, on financial misreporting. Our findings show that, rather than sitting on the sidelines, female CFOs can be more proactive and aggressively intervene to reduce firms' violations.

Second, prior research has found that CFO power has mixed results in terms of violations. On the one hand, increasing power magnifies the negative impact of female CFOs on violations; on the other hand, higher power persuades female CFOs to become overconfident and take more risks in their decision-making, resulting in more information disclosure violations. We extended the literature by measuring power in four dimensions, namely, structural power, ownership power, expert power, and prestige power. We showed that CFO power enhances female CFOs' ability to restrict the firm's opportunistic behavior, resulting in fewer information disclosure violations. This finding complements the study on the influence of managers in the firm's decision-making process.

Our results also have some practical implications. First, the sex ratio at birth in China demonstrates a major situation of “missing women” due to the strong son preference in Chinese culture. According to a research published by the World Economic Forum in 2018, China ranks 149th out of 149 nations in terms of Health and Survival gender gaps. Clearly, the enormous gender disparity in China can be explained not only by ethical, moral, or social fairness theories (Sun et al., 2019) but also by the economic benefits of the unique skills, experiences, and talents that women bring to such roles (Carter et al., 2010). By studying the effect of female CFO on information disclosure violations, we discovered that women's distinct morality and risk aversion can effectively limit a firm's susceptibility to information disclosure violations. Our results provide empirical evidence of women's positive contributions to firm, which will aid in the reduction of gender discrimination.

Second, our findings are useful to regulators. We showed that firms with female CFOs are less likely to engage in information violations because female CFOs are more ethical and risk-averse, providing archival evidence that regulators should be more cautious when reviewing the financial reports of male CFOs.

The remainder of this article presents the hypothesis, discusses the data and research methodology, analyzes the empirical results and robust results, and concludes the study.

## Hypothesis Development

### Female CFOs and Information Disclosure Violations

Traditional financial theory posits that in a perfect capital market with no friction and asymmetric information, managers may choose the optimal investments to maximize the firm's value, with managers' preferences having no influence on investment selection (Croson and Gneezy, 2009). In reality, the decision-maker's preferences and characteristics will have a role in a firm's investment selection due to the agent cost and asymmetric information. Women are less risk tolerant in general than their male counterparts, according to experimental economics and psychology research. Female CEOs and chairwomen are more likely to invest prudently and implement safer company strategies (Francis et al., 2015; Faccio et al., 2016). Because strategic decisions and information disclosure need a significant level of discretion, we can predict that female CFOs will use more conservative ways in information disclosure, resulting in fewer information disclosure violations.

According to behavioral finance, men are often more overconfident than women. Overconfident CEOs are also prone to overestimating investment returns and underestimating risks (Malmendier and Tate, 2008). Recent studies found that female CEOs are less likely than male executives to engage in acquisitions and issue loans (Huang and Kisgen, 2013). Because less overconfident agents may reduce risk to a level that suits their tastes after becoming executives, female executives who become CFOs would provide more conservative financial reports, resulting in fewer fake financial reports (Sun et al., 2019).

Additionally, women also hold fewer corporate board positions than their male counterparts. In our sample, women occupied 12.11% of board seats in China. When it comes to obtaining a new career, women will have a harder time than men (Faccio et al., 2016). According to data from the European Labor Force Survey, the average unemployment rate among women who previously held a managerial position is 3.9%, while this rate is only 2.7% for men. Phelps and Mason (1991) further documented that after leaving a managerial job, women tend to stay unemployed for longer periods than men. Women and men confront different levels of unemployment risk, which will influence their corporate tactics. More specifically, to reduce unemployment risk, women may opt to self-select into low-violation enterprises or lower firm violation once they have become CFOs.

Finally, the gender-ethics framework demonstrates that, in comparison with men, women are more concerned with ethical issues and adhere to higher moral norms (Croson and Gneezy, 2009). This may encourage increased transparency in financial reporting while also discouraging earnings management (Ho et al., 2015). Furthermore, the Sarbanes–Oxley Act holds senior executives personally liable for the accuracy and completeness of financial reports filed by their companies. As a result, female CFOs' conservative thinking and strong opposition to fraud are likely to strengthen compliance with the Sarbanes–Oxley Act and lower the likelihood of corporate information disclosure violations. Given the above analysis, we predict a negative association between the presence of a female CFO and a firm's violation of information disclosure.

**H1**: Firm with female CFOs will engage in less information disclosure violations.

### The Moderate Effects of CFO Power

Given that power is defined as the “capacity of individual actors to exert their will as means of pursing their goals” (Finkelstein, 1992), powerful CFOs can have a greater impact on corporate processes and outcomes. The more decision-making power female CFOs have, the more likely they are to influence the organization through their own risk-averse qualities, resulting in fewer violations in our case. From this perspective, we believe that CFO power enhances the female gender's effectiveness in reducing disclosure violations.

On the contrary, the approach/inhibition theory of power (Keltner et al., 2003) in social psychology posits that power, a fundamental element of human interaction, triggers the behavioral approach system, which leads those in positions of power to focus on the potential rewards of risky behavior while ignoring potential threats (Lewellyn and Muller-Kahle, 2012). Anderson and Galinsky (2006) discovered a link between individual risk taking and the existence of power. Additionally, they also found that power increases optimism in risk perception, which leads to an increase in the proclivity for risk-taking behavior (Anderson and Galinsky, 2006). Prior studies also suggest that powerful CEOs will lead to excessive risk taking (Lewellyn and Muller-Kahle, 2012; Sariol and Abebe, 2017). Following this assertion, power may seduce CFO into riskier behavior such as disclosure violations.

We investigated the moderate effect of CFO power on the link between CFO gender and disclosure violations because the role of CFO power is unclear. As a result, we proposed the following hypothesis:

***H2 (a)***: The negative relationship between female CFOs and information disclosure violations may be stronger when CFOs have more power.
***H2 (b)***: The negative relationship between female CFOs and information disclosure violations may be weaker when CFOs have more power.

### The Moderate Effect of External Monitoring

As suggested by Jensen and Meckling (1976), effective monitoring can restrain executive opportunities to capture personal benefits. External monitoring measures, if effective, can mitigate agency problems and create a good corporate governance environment. Furthermore, various studies have proven that excellent corporate governance policies have a positive impact on a company's current and future operating performance (Bhagat and Bolton, 2008). Good governance can increase a company's financial disclosure and transparency, resulting in lower debt and equity costs, as well as higher market valuations and accounting performance (González et al., 2021). Based on this line of reasoning, we presented the following hypothesis:

***H3***: The negative relationship between female CFOs and information disclosure violations is strengthened with effective external monitoring.

## Data and Methods

### Data

Our sample included all firms that were listed on the Shanghai and Shenzhen Stock Exchanges' A-share markets between 2003 and 2016. We chose 2003 as the beginning point because the ownership data were initially accessible in 2003. Because authorities need time to detect a firm's violations and disclose it, we selected 2016 as the end year rather than 2018. As a result, the year in our sample refers to the year in which the violation occurred, not the year in which the authorities disclosed or penalized. After excluding financial organizations (such as banks, insurance companies, and investment trusts) and firms with missing CFO characteristics, we finally received a sample of 20,258 observations representing 2,789 publicly traded companies. The table of industry-year distribution of the sample is provided in Appendix B in Supplementary Material. The data of information disclosure were hand-collected on the website of China's Securities and Regulatory Commission (CSRC). We gathered detailed information on disclosure violations, including the year of the violation, the exact categories of violation, the number of violations, and the authorities who impose the punishment; and we ended up with 2,405 violation records. Other CFO's personal information, accounting, and financial data were obtained from the China Stock Market and Accounting Research Database (CSMAR) and Wind Database.

### Variables Definition and Research Methodology

#### Dependent Variables

We used the following four different methods to assess information disclosure violations in our tests: *Violation, Number, False*, and *Improper*. If a firm breached the disclosure regulations in year *t, Violation* equals 1; otherwise, *Violation* equals 0. *Number* is the total number of breaches in year *t*. We also divided the violations into 2 categories, namely, false disclosure (*False*) and improper disclosure (*Improper*). The former refers to when a company's CFO makes incorrect or misleading claims (including false or misleading CSRC reports or financial statements). The latter refers to failing to disclose information on time, or disclosing information that contained a substantial omission, or wrongly using accounting judgment.

#### Independent Variables

*Gender* is the gender of the CFO and equals 1 if the CFO is a female and 0 otherwise. Since CSRC requires firms to provide the personal information of the entire management, we immediately identified the gender of a CFO. If the CFO has been replaced, *Gender* relates to the CFO with long tenure in year *t*. If the tenure in year *t* is the same (meaning that a new CFO joins the firm in July, which is just 36 observations in our sample), we referred to the CFO with long tenure during her stay in this firm. In robustness tests, the core results remain the same even if we exclude the 36 observations.

#### Control Variables

Following the information disclosure literature, we controlled for a vector of CFO, firm, and industry characteristics that may affect a firm's disclosure violation. The control variables include *Size* (firm's natural log of total assets), *Lev* (book value of long-term liabilities scaled by total assets), *ROA* (income before extraordinary items divided by total assets), *Loss* (dummy variable, and 1 means loss in net income), *Tobin's Q* (the sum of market value of equity and book value of liability, then scaled by total assets), *SOE* (dummy variable and 1 for state-owned firm), *List* (list year of the firm), *Big4* (dummy variable, and 1 indicates that financial report is audited by the big 4 audit firms), *Board* (board size and the number of board directors), *Indep* (proportion of independent directors), *Duality* (dummy variable, and 1 indicates that Chairman and CEO are the same person), *Age* (the natural log value of age of CFO), *Tenure* (the natural log value of monthly tenure of CFO), *Degree* (dummy variable, and 1 indicates that CFO has a master's of doctoral degree), *Major* (dummy variable, and 1 indicates that the college major of CFO is accounting or finance), *Abroad* (dummy variable, and 1 indicates that CFO has studied or worked abroad before), *PC* (dummy variable, and 1 implies that the CFO has previous or concurrent work experience in the government or a political position, such as a People's Representative or a Member of Chinese People's Political Consultative Conference). Besides that, we also included industry and year dummies to account for industry and year fixed effects.

#### Moderating Variables

##### CFO Power

Finkelstein (1992) proposed that management power originates from four dimensions, namely, structural power, ownership power, expert power, and prestige power. Following Lewellyn and Muller-Kahle (2012) and Lisic et al. (2016) in measuring CEO power, we first used two proxies for CFO structural power. We examined the CFO's title to see if he or she is also a director of the board (*Direct*). We also examined the CFO's relative compensation (*Dcomp*), defined as a dummy variable that equals 1 if CFO's total compensation (including salary, bonus, stock grants, and stock options) exceeds the industry-year median. We used whether CFO holds the outstanding share of the company (*Dshare*) to measure CFO ownership power, higher beneficial ownership gives the CFO greater power. As for expert power, we used CFO tenure (*Dtenure*) to measure, defined as a dummy variable that equals 1 if the CFO's tenure is longer than the industry-year median. For the CFO's prestige power, we adopted two variables outside, namely, board memberships (*Dboard*) and educational background (*Dedu*), for CFO's prestige power. *Dboard* is a dummy variable that equals 1 if the CFO sits on other corporate boards, and *Dedu* is a dummy variable that equals 1 if CFO has a master's or doctoral degree.

##### External Monitoring

Following Lu et al. (2015) and Dong et al. (2018), we employed external monitoring from institutional investor, auditor, analyst, the media, and local market to measure firms' corporate governance. Institutional investor (*Dinst*) is defined as a dummy variable that equals 1 if the institutional investors' shareholding is greater than the industry-year median. Auditor (*Daudit*) is also a dummy variable that equals 1 if the financial report has been audited by one of the big 4 auditor firms. We adopted analyst attention (*Danalyst*) for the monitoring from analysts, and *Danalyst* is a dummy variable that equals 1 if the number of analysts who follow the firm is greater than the industry-year median. We also used media attention (*Dmedia*) to assess media monitoring. *Dmedia* is a dummy variable that equals 1 if the firm's media coverage exceeds the industry-year median. For local market monitoring, we used two variables, namely, regional marketization (*DMI*) and local trust (*Dtrust*). *DMI* is a dummy variable that equals 1 if the province's marketization index (MI)^1^ where the firm is located is greater than the province-year median (Fan et al., 2013). *Dtrust* is also a dummy variable that equals 1 if the social trust index^2^ of the province in which the firm is located is greater than the province-year median. The detailed definitions of all the variables are presented in Appendix A in Supplementary Material.

#### Methodology

To examine the impact of CFO's gender on information disclosure violations, we used the following regression model:


(1) $$\begin{array}{l} Disclosure\;Violation=\;\alpha_{0}+\alpha_{1}\times Gender+Control+Ind\\ \;+Year+\mu \end{array}$$

We used the following empirical model to test the moderate effect of CFO power:


(2) $$\begin{array}{l} Disclosure\;Violation=\;\alpha_{0}+\alpha_{1}\times Gender+\alpha_{2}\times Ability\\ \;+\;\alpha_{3}\times Gender\times Ability\\ \;+\;Control+Ind+Year+\mu \end{array}$$

where *Ability* is a general measure for the six proxies, namely, *Direct, Dcomp, Dshare, Dtenure, Dboard*, and *Dedu*.

We used the following empirical model to test the moderate effect of external monitoring:


(3) $$\begin{array}{l} Disclosure\;Violation=\;\alpha_{0}+\alpha_{1}\times Gender+\alpha_{2}\times Governance\\ \;+\alpha_{3}\times Gender\times Governance+Control\\ \;+Ind+Year+\mu \end{array}$$

where *Governance* is a general measure for the six proxies, namely, *Dinst, Daudit, Danalyst, Dmedia, DMI*, and *Dtrust*.

Since the *Violation* is a dummy variable and *Number* is a discrete variable that is never below zero, we used the Probit model and Tobit model in regression to control the left-censored sample. At the same time, to control the robustness of results, we reported *t*-statistics that are computed using heteroskedasticity-robust standard errors clustered by firm and year (Petersen, 2009; Thompson, 2011).

## Empirical Results

### Summary Statistics

Panel A of Table 1 presents the sample's summary statistics. The mean of *Violation* for the entire sample is 0.119, implying that 11.9% of the companies in the study have been fined for information disclosure violations. Furthermore, the mean of *Number* is 0.167, which is higher than the median, showing that some companies have had several violations of information disclosure. The means of *False* and *Improper* are 0.059 and 0.107, respectively. The sum of the *False* and *Improper* means does not equal the mean of *Violation* suggests that some firms engage in both false and improper information violations. This finding suggests that the firm as a whole makes more improper disclosures than false disclosures. The mean of gender is 0.29, indicating 29.1% of CFOs are female and females are far more likely to be CFOs than CEOs or Chairman of the Board, as Feng and Johansson (2018) found that 95.9% of Chairman are male. To minimize the effect of outliers, we winsorized all continuous variables at the top and bottom 1% of each variable's distribution.

**Table 1:** Summary statistics and univariate comparisons.

	***N***	**P5**	**P25**	**Mean**	**Median**	**P75**	**P95**	**Std. dev**
**Panel A: Summary statistics**								
Violation	20,258	0	0	0	0.119	0	1	0.323
Number	20,258	0	0	0	0.167	0	2	0.531
False	20,258	0	0	0	0.059	0	1	0.236
Improper	20,258	0	0	0	0.107	0	1	0.309
Gender	20,258	0	0	0	0.291	1	1	0.454
Size	20,258	19.057	20.909	21.645	21.814	22.514	26.204	1.318
Lev	20,258	0	0	0.022	0.070	0.106	0.443	0.100
ROA	20,258	−0.283	0.015	0.040	0.042	0.074	0.241	0.073
Loss	20,258	0	0	0	0.109	0	1	0.312
Tobin's Q	20,258	0.152	0.560	0.728	0.702	0.844	1.532	0.231
SOE	20,258	0	0	0	0.491	1	1	0.500
List	20,258	1	4	8	8.960	13	22	5.734
Big4	20,258	0	0	0	0.062	0	1	0.241
Board	20,258	5	8	9	9.026	9	15	1.891
Indep	20,258	0.250	0.333	0.333	0.365	0.400	0.556	0.053
Duality	20,258	0	0	0	0.205	0	1	0.404
Age	20,258	3.466	3.714	3.807	3.806	3.912	4.127	0.145
Tenure	20,258	0.693	2.565	3.258	3.177	3.892	4.956	0.976
Degree	20,258	0	0	1	0.514	1	1	0.500
Major	20,258	0	1	1	0.754	1	1	0.430
Abroad	20,258	0	0	0	0.018	0	1	0.134
PC	20,258	0	0	0	0.284	1	1	0.451
	**Female CFOs**		**Male CFOs**		**Mean difference**	
	***N***	**Mean**	***N***	**Mean**	**Difference**	***T*** **-statistics**
**Panel B: Univariate comparisons**						
Violation	5,894	0.103	14,364	0.125	−0.022	4.293^***^
Number	5,894	0.143	14,364	0.176	−0.033	4.015^***^
False	5,894	0.048	14,364	0.064	−0.016	4.169^***^
Improper	5,894	0.094	14,364	0.112	−0.018	3.760^***^
	**Violation**	**Number**	**FALSE**	**Improper**	**Gender**	**Size**	**Lev**	**ROA**	**Loss**	**Tobin's Q**	**SOE**	**List**	**Big4**	**Board**	**Indep**	**Duality**	**Age**	**Tenure**	**Degree**	**Major**	**Abroad**
**Panel C: Correlation matrix**																					
Number	**0.859**																				
FALSE	**0.678**	**0.657**																			
Improper	**0.944**	**0.816**	**0.559**																		
Gender	**−0.031**	**−0.029**	**−0.029**	**−0.027**																	
Size	**−0.070**	**−0.059**	**−0.055**	**−0.074**	**−0.040**																
Lev	0.006	−0.003	−0.007	0.007	**−0.023**	**0.400**															
ROA	**−0.144**	**−0.138**	**−0.097**	**−0.141**	**0.027**	**0.106**	**−0.104**														
Loss	**0.141**	**0.140**	**0.093**	**0.140**	**−0.018**	**−0.133**	**0.033**	**−0.662**													
Tobin's Q	**0.018**	**0.021**	***0.016***	***0.016***	**0.049**	**−0.333**	**−0.196**	**0.066**	**0.066**												
SOE	**−0.043**	**−0.040**	**−0.044**	**−0.042**	**−0.073**	**0.188**	**0.187**	**−0.072**	**0.033**	**−0.214**											
List	**0.036**	**0.043**	***0.016***	**0.028**	**−0.024**	**0.264**	**0.187**	**−0.111**	**0.096**	**0.112**	**0.210**										
Big4	**−0.050**	**−0.038**	**−0.034**	**−0.057**	**−0.024**	**0.376**	**0.093**	**0.054**	**−0.045**	**−0.115**	**0.115**	**0.045**									
Board	**−0.033**	**−0.034**	**−0.024**	**−0.038**	**−0.043**	**0.249**	**0.129**	*0.011*	**−0.034**	**−0.193**	**0.266**	−0.005	**0.172**								
Indep	−0.005	0.000	0.000	−0.009	***0.015***	**0.177**	**0.032**	0.009	0.006	**0.111**	**−0.202**	**0.194**	**0.045**	**−0.238**							
Duality	**0.021**	**0.019**	**0.026**	0.010	**0.051**	**−0.098**	**−0.102**	**0.025**	***−0.015***	**0.090**	**−0.251**	**−0.125**	**−0.065**	**−0.155**	**0.092**						
Age	***−0.017***	−0.010	*−0.012*	**−0.020**	**0.060**	**0.191**	**0.039**	*0.012*	−0.011	*0.013*	**0.101**	**0.150**	**0.059**	**0.046**	**0.060**	**−0.037**					
Tenure	**−0.027**	**−0.019**	**−0.018**	**−0.033**	**0.034**	**0.190**	***0.016***	**0.046**	**−0.033**	**0.105**	**−0.110**	**0.154**	0.006	**−0.054**	**0.163**	**0.060**	**0.260**				
Degree	0.000	0.000	−0.006	−0.001	**−0.025**	**0.132**	**0.092**	**−0.043**	**0.022**	**−0.025**	**0.152**	**0.232**	**0.077**	**0.063**	**−0.078**	**−0.080**	**−0.077**	**−0.080**			
Major	***0.017***	**0.018**	**0.020**	0.007	**0.040**	**0.276**	**0.031**	**0.077**	**−0.037**	**0.220**	**−0.256**	**0.266**	−0.002	**−0.162**	**0.440**	**0.116**	**0.176**	**0.354**	**−0.033**		
Abroad	−0.007	−0.006	−0.007	−0.007	**−0.027**	**0.072**	0.001	**0.027**	**−0.023**	**0.021**	**−0.053**	***−0.016***	**0.084**	0.007	**0.032**	**0.033**	0.012	**0.046**	**0.054**	**0.083**	
PC	***−0.016***	***−0.015***	**−0.018**	−0.008	**−0.051**	**−0.221**	−0.009	**−0.070**	**0.027**	**−0.225**	**0.262**	**−0.248**	***0.013***	**0.170**	**−0.404**	**−0.116**	**−0.130**	**−0.322**	**0.037**	**−0.909**	**−0.059**

This table presents summary statistics and univariate comparisons among the main variables during the period 2003–2016. Panel A provides the descriptive statistics, and columns 1–8 present the number, 5th percentile, 25th percentile, mean, median, 75th percentile, 95th percentile, and standard deviation for each variable, respectively. Panel B provides the univariate comparisons and significance at the 10, 5, and 1% levels and are indicated by ^*^, ^**^, and ^***^, respectively. Panel C presents the Pearson's correlation for the dependent and independent variables. Bold, bold-italicized, and italicized correlations represent statistical significance at 1, 5, and 10%, respectively. All the variables are defined in Appendix A in Supplementary Material.

Panel B presents univariate comparisons of disclosure violations between male and female CFOs. We found that the means of *Violation* for female and male CFOs are 0.103 and 0.125, respectively. The mean difference of−0.022 is significant at the 1% level. We also found that the means of *Number, False*, and *Improper* are all lower for female CFOs (significant at the 1% level). These univariate comparisons support our hypothesis that female CFOs are more cautious about disclosing information than their male counterparts.

Panel C presents the correlation coefficients. Consistent with H1, the gender of CFOs (*Gender*) is negatively correlated with four different measures of violations (i.e., *Violation, Number, False*, and *Improper*), suggesting that female CFOs are related to a lower level of information violations. In addition, panel C shows that the correlations among the control variables are not very high, indicating that multicollinearity should not be a major concern.

### Base Results

To investigate the relation between CFO gender and information disclosure violation, we started by regressing our measures of violation on CFO gender and other determinants of information disclosure violation We reported our baseline regression results in Table 2. As noted earlier, we used four different measures of violation in our tests, namely, *Violation, Number, False*, and *Improper*.

**Table 2:** Female CFOs and information disclosure violations.

	**(1)**	**(2)**	**(3)**	**(4)**
	**Violation**	**Number**	**False**	**Improper**
Gender	−0.122^***^	−0.035^***^	−0.163^***^	−0.103^**^
	(−2.98)	(−2.70)	(−3.27)	(−2.46)
Size	−0.069^***^	−0.019^***^	−0.065^***^	−0.065^***^
	(−3.34)	(−2.87)	(−2.58)	(−3.09)
Lev	0.405^*^	0.029	0.248	0.455^**^
	(1.85)	(0.44)	(0.96)	(2.05)
ROA	−1.681^***^	−0.546^***^	−1.531^***^	−1.644^***^
	(−5.87)	(−5.25)	(−4.25)	(−6.07)
Loss	0.244^***^	0.130^***^	0.201^***^	0.244^***^
	(4.87)	(6.08)	(3.13)	(4.84)
Tobin's Q	0.270^***^	0.101^***^	0.118	0.299^***^
	(3.43)	(3.72)	(1.26)	(3.71)
SOE	−0.188^***^	−0.057^***^	−0.179^***^	−0.185^***^
	(−4.11)	(−3.48)	(−3.21)	(−3.90)
List	0.021^***^	0.007^***^	0.013^**^	0.019^***^
	(4.99)	(4.30)	(2.45)	(4.53)
Big4	−0.221	−0.019	−0.152	−0.367^***^
	(−1.59)	(−0.59)	(−0.75)	(−3.31)
Board	−0.003	−0.003	−0.004	−0.012
	(−0.26)	(−0.80)	(−0.24)	(−0.97)
Indep	−0.321	−0.097	−0.735^*^	−0.325
	(−0.91)	(−0.85)	(−1.70)	(−0.88)
Duality	0.086^**^	0.024	0.115^**^	0.032
	(2.01)	(1.61)	(2.14)	(0.73)
Age	0.033	0.025	0.051	0.020
	(0.24)	(0.59)	(0.30)	(0.14)
Tenure	−0.043^***^	−0.013^**^	−0.047^**^	−0.042^**^
	(−2.61)	(−2.11)	(−2.50)	(−2.44)
Degree	−0.023	−0.007	−0.038	−0.021
	(−0.58)	(−0.50)	(−0.76)	(−0.55)
Major	0.002	−0.053	−0.076	−0.063
	(0.01)	(−0.54)	(−0.37)	(−0.35)
Abroad	−0.001	−0.008	−0.070	0.021
	(−0.00)	(−0.21)	(−0.45)	(0.16)
PC	0.058	0.033	0.075	0.058
	(0.66)	(0.93)	(0.64)	(0.64)
Constant	0.440	0.576^***^	0.098	0.461
	(0.64)	(2.82)	(0.11)	(0.67)
Year and industry	Control	Control	Control	Control
*N*	20,258	20,258	20,258	20,258
Pseudo *R*^2^	0.061	0.028	0.054	0.063

This table presents the results of the impact of female CFOs on disclosure violations. In Regression (1), the dependent variable is Violation, where Violation is an indicator variable that takes the value of 1 if the firm has been penalized for information violation in year t, and 0 otherwise. In Regression (2), the dependent variable is Number, where Number is defined as the total amount that firm has engaged in information violation in year t. In Regressions (3) and (4), the dependent variables are indicators denoting whether the firm engaged in false disclosure or improper disclosure. Regressions (1), (3), and (4) are run using Probit models, while Regression (2) is run using Tobit model. Female CFO is an indicator variable that takes the value of 1 if the CFO is a woman, and 0 otherwise. Control variables are defined in Appendix A in Supplementary Material. T-values, adjusted for heteroskedasticity and clustering at the firm and year levels, are reported in parentheses below the coefficients. ^*^, ^**^, and ^***^ indicate statistical significance levels of 10, 5, and 1%, respectively.

*Violation* is the dependent variable in Regression (1). Regression (1) is a probit regression with standard errors clustered at the firm and year level. According to the results of Regression (1), firms led by female CFOs are much less likely to be involved in violations than enterprises led by male CFOs. After controlling for several other determinants of violations, the coefficient of female CFO indicates that the violation of firms run by female CFOs is 0.122 lower on average than the violation of firms controlled by male CFOs. This difference appears to be statistically significant and economically significant. This finding provides supportive evidence that female CFOs are more cautious when it comes to information disclosure than their male counterparts.

In terms of the control variables of firm's performance, we found that the coefficients on *Size* and *SOE* are negative and significant, indicating that large or state-owned firms are less likely to commit disclosure violations because they are subject to more stringent investor and regulatory oversight. The coefficients on *Lev* and *Loss* are both negative and significant, which is in line with our expectations. Firms with higher debt and worse profitability are prone to participate in disclosure violations in order to meet the debt covenant requirements and analysts' forecasts. The coefficients on *Tobin's Q* and *List* are both positive and significant, indicating that firms with more investment opportunities and a longer list history are more likely to commit information disclosure violations.

In terms of the control variables of corporate governance, the coefficient on *Duality* is positive and significant, but the results of *Big4, Indep*, and *board* are not significant, indicating that the company's governance mechanisms have played only a partial role in information disclosure monitoring. For the control variables of CFO's personal characteristics, *Tenure* is inversely related to information disclosure violations, indicating that as the CFO obtains more experience during her tenure, the likelihood of violations decreases.

In Regression (2) of Table 2, we used *Number* as the dependent variable, and the control variables are the same as in Regression (1). We employed a panel Tobit model with standard errors clustered at the firm and year level. Consistent with the results for *Violation*, we found that *Gender* is statistically significant with a coefficient of−0.035, indicating that female CFOs are more conservative than their male colleagues. With regard to the control variables, we found that *Size, ROA, SOE*, and *Tenure* are all negative and significant, while *Loss, Tobin's Q*, and *List* are positive and significant, which is consistent with our expectations and prior findings.

In Regressions (3) and (4) of Table 2, we presented results in which the specifications are similar to that in Regressions (1) and (2), except that we used *False* and *Improper* as the dependent variables and found that *Gender* is negatively and significantly related with *False* and *Improper*. The coefficients of control variables are comparable with those results reported in Regressions (1) and (2).

In summary, our findings in Table 2 revealed that female CFOs are less likely to violate information disclosure laws on all four metrics. These findings corroborate our hypothesis that female CFOs are more cautious and risk-averse when it comes to information disclosure than male CFOs.

### Moderate Effect

#### CFO Power

Table 3 presents our results regarding the moderate effect of CFO power. In columns (1)–(6) from panel A to panel D, we found significantly negative coefficients on *Gender* × *Ability*, which support our first hypothesis that CFO power significantly strengthens the effectiveness of female CFO in reducing the incidence of disclosure violation. Powerful female CFOs can exercise considerable influence on information disclosure; therefore, their risk-averse trait can play a greater role in reducing violations.

**Table 3:** CFO power, gender, and information disclosure violations.

	**(1) Ability = Direct**	**(2) Ability = Dcomp**	**(3) Ability = Dshare**	**(4) Ability = Dtenure**	**(5) Ability = Dboard**	**(6) Ability = Dedu**
	**Structural power**		**Ownership power**	**Expert power**	**Prestige power**	
**Panel A: Y** **=** **Disclosure**						
Gender	−0.121	−0.062	−0.186^**^	−0.121	−0.101	−0.091
	(−1.36)	(−0.59)	(−2.38)	(−1.32)	(−1.11)	(−0.92)
Ability	0.152^*^	0.007	−0.088	−0.376^***^	−0.121^**^	−0.268^***^
	(1.65)	(0.09)	(−0.56)	(−4.01)	(−2.09)	(−3.58)
Gender × Ability	−0.354^**^	−0.329^**^	−0.859^**^	−0.402^**^	−0.239^**^	−0.352^**^
	(−2.19)	(−2.27)	(−2.31)	(−2.28)	(−2.12)	(−2.57)
Control variable	Control	Control	Control	Control	Control	Control
Year and industry	Control	Control	Control	Control	Control	Control
*N*	20,258	20,258	20,258	20,258	20,258	20,258
Pseudo *R*^2^	0.062	0.062	0.061	0.061	0.062	0.061
**Panel B: Y** **=** **Number**						
Gender	−0.018	−0.009	−0.030^**^	−0.019	−0.013	−0.018
	(−1.20)	(−0.49)	(−2.31)	(−1.10)	(−0.71)	(−0.88)
Ability	0.024	0.002	−0.030	−0.057^***^	−0.021^*^	−0.057^***^
	(1.26)	(0.14)	(−1.36)	(−3.92)	(−1.87)	(−3.82)
Gender × Ability	−0.057^**^	−0.049^**^	−0.058^*^	−0.042^*^	−0.040^**^	−0.039^*^
	(−2.15)	(−2.03)	(−1.85)	(−1.87)	(−2.07)	(−1.75)
Control variable	Control	Control	Control	Control	Control	Control
Year and industry	Control	Control	Control	Control	Control	Control
N	20,258	20,258	20,258	20,258	20,258	20,258
Pseudo *R*^2^	0.031	0.031	0.030	0.030	0.029	0.030
**Panel C: Y** **=** **False**						
Gender	−0.171	−0.037	−0.247^**^	−0.186	−0.152	−0.172
	(−1.43)	(−0.26)	(−2.30)	(−1.52)	(−1.24)	(−1.33)
Ability	0.147	−0.003	0.083	−0.319^**^	−0.071	−0.360^***^
	(1.12)	(−0.03)	(0.39)	(−2.39)	(−0.95)	(−3.53)
Gender × Ability	−0.479^**^	−0.598^***^	−1.638^***^	−0.529^**^	−0.296^*^	−0.393^**^
	(−2.08)	(−2.92)	(−2.61)	(−2.09)	(−1.92)	(−1.97)
Control variable	Control	Control	Control	Control	Control	Control
Year and industry	Control	Control	Control	Control	Control	Control
*N*	20,258	20,258	20,258	20,258	20,258	20,258
Pseudo *R*^2^	0.054	0.055	0.055	0.053	0.053	0.053
**Panel D: Y** **=** **Improper**	
Gender	−0.098	−0.074	−0.171^**^	−0.108	−0.090	−0.093
	(−1.06)	(−0.70)	(−2.11)	(−1.15)	(−0.97)	(−0.91)
Ability	0.159^*^	−0.018	−0.136	−0.473^***^	−0.130^**^	−0.301^***^
	(1.69)	(−0.22)	(−0.81)	(−4.84)	(−2.13)	(−3.92)
Gender × Ability	−0.374^**^	−0.264^*^	−0.795^**^	−0.373^**^	−0.224^*^	−0.312^**^
	(−2.22)	(−1.76)	(−2.03)	(−2.02)	(−1.90)	(−2.22)
Control variable	Control	Control	Control	Control	Control	Control
Year and industry	Control	Control	Control	Control	Control	Control
N	20258	20,258	20,258	20,258	20,258	20,258
Pseudo *R*^2^	0.063	0.062	0.062	0.063	0.062	0.063

This table presents the moderate results of CFO power on the relationship between female CFOs and disclosure violations. From panel A to panel D, the dependent variables are Violation, Number, False, and Improper, respectively, where the definitions of dependent variables, independent variable, and control variables are the same as in Table 2. In columns (1) and (2), we adopted Direct and Dcomp to measure CFO structural power, where Direct is an indicator variable that takes the value of 1 if CFO is also the director of the board, and 0 otherwise; Dcomp is an indicator variable equals to 1 if the CFO's total compensation is higher than the industry-year median, and 0 otherwise. We used Dshare to measure CFO ownership power where Dshare is an indicator variable that equals 1 if CFO holds the outstanding share of the firm, and 0 otherwise. As for expert power, we used Dtenure to measure expert power, where Dtenure is defined as a dummy variable and equals 1 if the tenure of CFO is longer than the industry-year median. We finally used Dboard and Dedu for CFO's prestige power. Dboard is a dummy variable and equals 1 if CFO holds directorates on other corporate boards, and Dedu is a dummy variable and equals 1 if CFO holds a master or doctoral degree. Panels A, C, and D are run using Probit models, while panel B is run using Tobit model. All the variables are defined in Appendix A in Supplementary Material. T-values, adjusted for heteroskedasticity and clustering at the firm and year levels, are reported in parentheses below the coefficients. ^*^, ^**^, and ^***^ indicate statistical significance levels of 10, 5, and 1%, respectively.

#### External Monitoring

Table 4 presents our results regarding the moderate effect of external monitoring. The results from panel A–panel D in Table 4 show that the interaction coefficients *Gender* × *Governance* through columns (1)–(6) are constantly negative and significant, implying that the negative association between female CFOs and information disclosure violation is more pronounced for firms with stronger external monitoring. With the help of effective external monitoring, the female CFO can better reduce firms' information violations.

**Table 4:** External monitoring, gender, and information disclosure violation.

	**(1) Ability = *Dinst***	**(2) Ability= *Daudit***	**(3) Ability = *Danalyst***	**(4) Ability = *Dmedia***	**(5) Ability = DMI**	**(6) Ability = *Dtrust***
	**Institutional investor**	**Auditor**	**Analyst**	**Media**	**Local market**	
**Panel A: Y** **=** **Disclosure**						
Gender	0.013	−0.205^***^	−0.034	−0.093	0.095	0.067
	(0.13)	(−2.65)	(−0.35)	(−0.95)	(0.60)	(0.50)
Governance	−0.028	−0.911^***^	−0.556^***^	−0.038	−0.222^**^	−0.105
	(−0.45)	(−2.76)	(−7.71)	(−0.54)	(−2.16)	(−1.10)
Gender × Governance	−0.409^***^	−1.553^**^	−0.419^***^	−0.255^**^	−0.467^**^	−0.425^***^
	(−3.55)	(−2.35)	(−3.30)	(−2.11)	(−2.53)	(−2.59)
Control variable	Control	Control	Control	Control	Control	Control
Year and industry	Control	Control	Control	Control	Control	Control
N	20,258	20,258	20,258	20,258	20,258	20,258
Pseudo *R*^2^	0.063	0.063	0.063	0.063	0.062	0.064
**Panel B: Y=** **Number**						
Gender	−0.006	−0.032^**^	−0.010	−0.013	0.022	0.011
	(−0.27)	(−2.42)	(−0.40)	(−0.70)	(0.60)	(0.38)
Governance	−0.015	−0.088^**^	−0.106^***^	−0.007	−0.037^*^	−0.027
	(−1.22)	(−2.49)	(−6.92)	(−0.59)	(−1.67)	(−1.34)
Gender × Governance	−0.046^**^	−0.052	−0.041^*^	−0.040^**^	−0.077^**^	−0.061^*^
	(−2.27)	(−1.41)	(−1.72)	(−2.03)	(−1.97)	(−1.86)
Control variable	Control	Control	Control	Control	Control	Control
Year and industry	Control	Control	Control	Control	Control	Control
N	20,258	20,258	20,258	20,258	20,258	20,258
Pseudo *R*^2^	0.032	0.031	0.030	0.031	0.031	0.030
**Panel C: Y=** **False**						
Gender	−0.125	−0.277^***^	−0.119	−0.106	0.042	−0.029
	(−0.87)	(−2.63)	(−0.93)	(−0.76)	(0.19)	(−0.16)
Governance	−0.086	−0.775	−0.534^***^	−0.006	−0.032	−0.071
	(−1.03)	(−1.51)	(−5.34)	(−0.06)	(−0.22)	(−0.55)
Gender × Governance	−0.302^*^	−1.972^*^	−0.414^**^	−0.402^**^	−0.481^*^	−0.410^*^
	(−1.87)	(−1.75)	(−2.37)	(−2.28)	(−1.88)	(−1.83)
Control variable	Control	Control	Control	Control	Control	Control
Year and industry	Control	Control	Control	Control	Control	Control
N	20,258	20,258	20,258	20,258	20,258	20,258
Pseudo *R*^2^	0.055	0.055	0.056	0.056	0.055	0.055
**Panel D:** ***Y*****=** ***Improper***						
Gender	0.033	−0.195^**^	−0.005	−0.085	0.066	0.083
	(0.31)	(−2.43)	(−0.05)	(−0.84)	(0.41)	(0.60)
Governance	0.008	−1.250^***^	−0.536^***^	−0.080	−0.298^***^	−0.127
	(0.13)	(−4.35)	(−7.42)	(−1.11)	(−2.86)	(−1.28)
Gender × Governance	−0.408^***^	−1.517^**^	−0.445^***^	−0.238^*^	−0.415^**^	−0.413^**^
	(−3.41)	(−2.00)	(−3.39)	(−1.89)	(−2.18)	(−2.43)
Control variable	Control	Control	Control	Control	Control	Control
Year and industry	Control	Control	Control	Control	Control	Control
N	20,258	20,258	20,258	20,258	20,258	20,258
Pseudo *R*^2^	0.065	0.064	0.065	0.064	0.065	0.064

The moderate effects of external monitoring on the relationship between female CFOs and disclosure violations are presented in this table. The dependent variables in this table are Violation, Number, False, and Improper, with the same definitions of dependent variables, independent variables, and control variables as in Table 2. We used Dinst to measure monitor from institutional investors in column (1), where Dinst is a dummy variable that equals 1 if institutional investors' shareholding exceeds the industry-year median. We used Daudit to measure monitor from auditor in column (2), where Daudit is also a dummy variable that equals 1 if the financial report is audited by one of the big 4 auditor firms. In column (3), we adopted Danalyst for the monitoring from analyst, where Danalyst is a dummy variable and equals 1 if the number of analysts who follow the firm is greater than the industry-year median. In column (4), we adopted Dmedia for the monitoring from media, where Dmedia is a dummy variable and equals 1 if the firm's media coverage is greater than the industry-year median. In columns (5) and (6), we employed DMI and Dtrust for the monitoring from local market where DMI is a dummy variable and equals 1 if the marketization index (MI) of the province (Fan et al., 2013) where firm locate is greater than the province-year median. And Dtrust is also a dummy variable and equals 1 if the social trust index of the province where firm locate is greater than the province-year median. Panels A, C, and D are run using Probit models, while panel B is run using Tobit model. All the variables are defined in Appendix A in Supplementary Material. T-values, adjusted for heteroskedasticity and clustering at the firm and year levels, are reported in parentheses below the coefficients. ^*^, ^**^, and ^***^ indicate statistical significance levels of 10, 5, and 1%, respectively.

### Robustness Tests

#### Propensity Score Matching

Table 2 shows, however, that firm and CFO characteristics are highly connected with disclosure violations, implying that non-random selection is a possibility. To address sample selection problems, we used a PSM procedure to see if there are any noticeable differences in the characteristics of firms led by female CFOs vs. firms led by male CFOs. We defined firms with female CFOs as the treatment group and firms with male CFOs as the control group. To conduct a one-to-one PSM matching, we used control variables in the Regression (1), including *Size, Lev, ROA, Loss, Tobin's Q, SOE, List, Big4, Board, Indep, Duality, Age, Tenure, Degree, Major, Abroad, PC, Industry*, and *Year*.

After matching, the treatment and control groups appear to be nearly indistinguishable in terms of firm and CFO characteristics, as shown in panel A1 of Table 5. This test further confirmed the validity of our matching strategy. The means of *Violation* after matching are 0.103 and 0.126 for the treatment and control groups, respectively, and the difference is significant at the 1% level, suggesting that firms with male CFOs are more likely involved in information violation. The results are qualitatively similar for *Number, False*, and *Improper*. These provide additional evidence that our main findings are robust to alternative model specifications.

**Table 5:** Robust checks.

	**Pre-matching**			**Post-matching**		
	**Treatment**	**Control**	**Difference in means (t-statistic)**	**Treatment**	**Control**	**Difference in means (t-statistic)**
**Panel A: Propensity score matching**						
**Panel A1: Univariate comparisons**						
Violation	0.103	0.125	4.293^***^	0.103	0.126	3.845^***^
Number	0.143	0.176	4.015^***^	0.143	0.182	3.962^***^
False	0.048	0.064	4.169^***^	0.048	0.064	3.757^***^
Improper	0.094	0.112	3.760^***^	0.094	0.113	3.359^***^
Size	21.719	21.853	−10.30^***^	21.719	21.718	0.100
Lev	0.066	0.072	−5.70^***^	0.066	0.066	0.100
ROA	0.045	0.040	5.90^***^	0.045	0.045	−0.100
Loss	0.100	0.113	−4.20^***^	0.100	0.098	0.500
Tobin's Q	0.691	0.706	−6.40^***^	0.691	0.692	−0.300
SOE	0.433	0.516	−16.70^***^	0.433	0.432	0.100
List	8.694	9.070	−6.50^***^	8.694	8.622	1.300
Big4	0.052	0.066	−6.30^***^	0.052	0.055	−1.300
Board	8.896	9.080	−9.80^***^	8.896	8.937	−2.200
Indep	0.365	0.364	2.60^***^	0.365	0.365	0.500
Duality	0.241	0.191	12.20^***^	0.241	0.243	−0.500
Age	3.817	3.801	10.90^***^	3.817	3.817	−0.100
Tenure	3.227	3.157	7.20^***^	3.227	3.208	1.900
Degree	0.495	0.522	−5.40^***^	0.495	0.505	−2.000
Major	0.782	0.743	9.10^***^	0.782	0.780	0.500
Abroad	0.012	0.021	−6.60^***^	0.012	0.014	−1.700
PC	0.246	0.299	−11.90^***^	0.246	0.248	−0.400
	**(1)**	**(2)**	**(3)**	**(4)**
	**Violation**	**Number**	**False**	**Improper**
**Panel A2: Multivariate regression analysis**				
Gender	−0.129^***^	−0.039^***^	−0.160^***^	−0.115^**^
	(−2.93)	(−2.69)	(−3.00)	(−2.52)
Control variables	Control	Control	Control	Control
Year and industry	Control	Control	Control	Control
*N*	11786	11786	11786	11786
Pseudo *R*^2^	0.071	0.034	0.064	0.074
	**(1)**	**(2)**	**(3)**	**(4)**	**(5)**
	**Gender**	**Violation**	**Number**	**False**	**Improper**
**Panel B: Selection bias and Heckman two-stage self-selection models**					
Gender		−0.092^***^	−0.044^***^	−0.108^***^	−0.105^**^
		(−2.84)	(−2.61)	(−3.22)	(−2.37)
Ratio	4.850^***^				
	(7.29)				
IInvmr	5.017^***^	0.532	0.894	0.387	0.274
	(7.98)	(0.43)	(0.64)	(0.75)	(0.81)
Control variables	Control	Control	Control	Control	Control
Year and industry	Control	Control	Control	Control	Control
*N*	11,786	11,786	11,786	11,786	11,786
Pseudo *R*^2^		0.071	0.034	0.064	0.074
Likelihood ratio	238.32^***^				
**Panel C: Event study and DID test**				
Post	−0.158	−0.203	−0.087	−0.055^*^
	(−1.11)	(−1.13)	(−0.57)	(−1.84)
Up	0.075	0.091	0.204	0.036
	(0.31)	(0.29)	(0.84)	(0.52)
Up × Post	−0.528^*^	−0.816^*^	−0.480^*^	−0.106
	(−1.91)	(−1.96)	(−1.71)	(−1.58)
Down	−1.260^***^	−1.528^**^	−1.341^***^	−0.178^***^
	(−3.51)	(−2.43)	(−3.52)	(−5.15)
Down × Post	1.008^***^	1.033^*^	1.085^**^	0.136^***^
	(2.69)	(1.94)	(2.57)	(2.98)
Control variables	Control	Control	Control	Control
Year and industry	Control	Control	Control	Control
*N*	3,016	3,016	3,016	3,016
Pseudo *R*^2^	0.073	0.083	0.080	0.029
	**(1)**	**(2)**	**(3)**	**(4)**
	**Violation**	**Number**	**False**	**Improper**
**Panel D: Control the gender of CEO and chairman of the board**				
**Subsample: female CEO**				
Gender	−0.695^**^	−0.123^**^	−0.577^**^	−0.769^**^
	(−2.48)	(−2.38)	(−2.05)	(−2.55)
Control variable	Control	Control	Control	Control
Year and industry	Control	Control	Control	Control
*N*	1,119	1,119	1,119	1,119
Pseudo *R*^2^	0.015	0.005	0.009	0.017
**Subsample: male CEO**				
Gender	−0.180^**^	−0.027^**^	−0.271^**^	−0.152^**^
	(−2.26)	(−2.05)	(−2.46)	(−2.06)
Control variable	Control	Control	Control	Control
Year and industry	Control	Control	Control	Control
N	18,939	18,939	18,939	18,939
Pseudo *R*^2^	0.001	0.000	0.002	0.001
**Subsample: female chairs of the board**				
Gender	−0.698^**^	−0.124^**^	−1.078^**^	−0.592^**^
	(−2.05)	(−2.13)	(−2.43)	(−2.26)
Control variable	Control	Control	Control	Control
Year and industry	Control	Control	Control	Control
*N*	914	914	914	914
Pseudo *R*^2^	0.014	0.006	0.026	0.010
**Subsample: male chairs of the board**				
Gender	−0.189^**^	−0.029^**^	−0.250^**^	−0.175^**^
	(−2.44)	(−2.27)	(−2.32)	(−2.18)
Control variable	Control	Control	Control	Control
Year and industry	Control	Control	Control	Control
**Panel E: Different sample period**				
**Sample period 2003–2015**				
Gender	−0.227^***^	−0.036^***^	−0.335^***^	−0.196^**^
	(−2.78)	(−2.59)	(−3.06)	(−2.30)
Control variable	Control	Control	Control	Control
Year and industry	Control	Control	Control	Control
N	18,060	18,060	18,060	18,060
Pseudo *R*^2^	0.058	0.027	0.050	0.061
**Sample period 2003–2014**				
Gender	−0.276^***^	−0.042^***^	−0.376^***^	−0.253^***^
	(−3.16)	(−2.83)	(−3.18)	(−2.78)
Control variable	Control	Control	Control	Control
Year and industry	Control	Control	Control	Control
*N*	16,028	16,028	16,028	16,028
Pseudo *R*^2^	0.058	0.028	0.050	0.061
	**(1)**	**(2)**	**(3)**	**(4)**
	**Violation**	**Number**	**False**	**Improper**
**Panel F: Exogenous shock**				
**Before the issuance of regulation 2003–2006**				
Gender	−0.323^*^	−0.026^*^	−0.536^**^	−0.312^*^
	(−1.75)	(−1.74)	(−2.03)	(−1.82)
Control variable	Control	Control	Control	Control
Year and industry	Control	Control	Control	Control
*N*	3,801	3,807	3,746	3,801
Pseudo *R*^2^	0.139	0.079	0.096	0.153
**After the issuance of regulation 2007–2016**				
Gender	−0.210^**^	−0.035^**^	−0.299^***^	−0.178^**^
	(−2.57)	(−2.52)	(−2.69)	(−2.09)
Control variable	Control	Control	Control	Control
Year and industry	Control	Control	Control	Control
*N*	16,451	16,451	16,451	16,451
Pseudo *R*^2^	0.050	0.023	0.048	0.051

This table presents the results of several robustness checks. In panel A, we used Size, Lev, ROA, Loss, Tobin's Q, SOE, List, Big4, Board, Indep, Duality, Age, Tenure, Degree, Major, Abroad, PC, Industry, and Year to conduct a one-to-one PSM matching. Panel A1 presents univariate comparisons between treatment and control groups, while panel A2 presents the regression results using the post-matching sample. Panel B uses Heckman two-stage self-selection models to control for the fact that the gender of CFO is an endogenous choice. Panel C uses CFO transition as an event shock and construct a DID test by comparing the different effects of gender change on violations. In panel D, we split the full sample into female CEOs vs. male CEOs and female chairs vs. male chairs, respectively, to rule out the possibility that the main results are caused by the risk-aversion of CEO/chairs and their tendency to hire female CFO. In panel E, we replaced the ending period with 2014 and 2015 to alleviate the concern that violations in 2016 may be partially discovered. We used the issuance of “Rule of information disclosure for Listed Companies” by CSRC as an exogenous shock in panel F and test the results separately before and after the issuance. All variables are defined in Appendix A in Supplementary Material. The t-statistics (in parentheses) are based on standard errors clustered by the firm and year. ^***^, ^**^, and ^*^ indicate 1, 5, and 10% significance (two-tailed), respectively.

We also ran regressions using the treatment and control groups as samples, as shown in Table 2. Panel A2 of Table 5 shows that the regression results and the coefficients on *Gender* are all negative and significant under our different measures of violation, namely, *Violation, Number, False* and *Improper*, further indicating that the main regression result is robust.

#### Selection Bias

The primary focus of this article is on whether female CFOs can prevent corporations from violating information disclosure laws. However, firms pick the gender of their CFO, and this underlying choice may potentially affect the inference of the regression results in Table 2. To address the potential concern of selection bias, we adopted two-stage self-selection models. We used the sex ratio in the firm's province as an instrument to evaluate a firm's tendency to hire a female CFO in the first stage, in addition to control the same variables as in Table 2. For each firm, we calculated the predicted probability of hiring a female CFO from the fitted values of the Probit model.

In the second stage, we used the predicted probability to generate an inverse Mill's ratio to proxy for the likelihood of a female CFO, which is indicated in the regression results as “Heckman's lambda.” Regression results in panel B of Table 5 show that the results are qualitatively unchanged, suggesting that selection bias is not a serious concern.

#### DID Test Using a Sample of CFO Transition

In the previous section, we showed that there is a negative relationship between female CFOs and disclosure violations controlling for other factors that have been shown to affect violations. The negative relationship may be caused by unobservable invisible variables. To partially alleviate the endogeneity caused by unobservable variables, we employed a sample of enterprises that are transitioning from male to female CEOs or vice versa. Focusing on transition firms allows us to compare the risk-taking of the same firms, as led by CFOs of different genders. We used the transition of CFO as an event shock and employed the event study and DID approach to determine the effect of the CFO's gender on the firm's violation.

We began by identifying the samples that have seen a CFO change. To avoid the tenure being too short or too long for CFO to play her role in refraining from disclosure violation, we required a 2-year tenure before and after the change, i.e., the balancing event window is 4 years. We also tested the relationship between female CFO and violations in a 6-year balancing event window, and the results are almost the same as in the 4-year window. Hence, we kept the sample with the 4-year event window and eliminated the firm that has never changed CFO during the research period or firms with less than the 4-year event window.

By comparing CFOs' gender before and after transition, we divided our event study sample into three groups. Group 1 is the control group that has no change of gender before and after the CFO transition, i.e., the CFO is changed from a male CFO to a male CFO or from a female CFO to a female CFO. Group 2 is made up of firms that have switched from a male to a female CFO, while Group 3 is made up of those that have switched from a female to a male CFO. We then constructed variables *up* and *down* to distinguish the different groups and use the following empirical model to test our hypothesis:


(4) $$\begin{array}{l} Disclosure\;Violation=\;\alpha_{0}+\alpha_{1}\times Post+\alpha_{2}\times Up+\alpha_{3}\times Down\\ \;+\alpha_{4}\times Up\times Post+\alpha_{5}\times Down\\ \;\times Post+Control+Ind+Year+\mu \end{array}$$

where *Post* is a dummy variable that equals 1 if a year is after the CFO transition year, and 0 if a year is before the transition year. *Up* is a dummy variable that equals 1 if the transition is from a male CFO to a female CFO and 0 if the transition is from a female CFO to a male CFO or the gender stays the same before and after the transition. *Down* is a dummy variable like *Up* that equals 1 if the transition is from a female CFO to a male CFO and 0 otherwise. *Up* × *Post* and *Down* × *Post* are our primary variables of interest.

If the base results are robust that a female CFO can refrain from violations, we can see the coefficient of *Up* × *Post* is positive, while *Down* × *Post* is negative, indicating that when a firm changes from a male (or female) CFO to a female (or male) CFO, it is less (more) likely to be involved in disclosure violations after the transition.

In Panel C of Table 5, the interaction coefficient of *Up* × *Post* is negative and both economically and statistically significant, while the coefficient of *Down* × *Post* is positive for all four measures of information disclosure violation. This shows that the transition from a male CFO to a female CFO has a statistically significant and economically meaningful impact on disclosure violations. Specifically, the coefficient on *Up* × *Post* is −0.528 and is significant at the 10% level for the variable *Violation*, indicating that *Violation* is about −0.528 lower for the post-transition period (under the control of no gender change) as compared with the pre-transition period, comparing to an increase of 1.008 of *Violation* due to change from male CFO to female CFO. This finding provides supportive evidence that CFO transitions are associated with changes in corporate risk-taking. In particular, transitions from male to female CEOs are associated with a reduction in information violations. Female CFOs are more conservative in information disclosure decision-making than their male counterparts.

#### Control the Gender of CEO and Chairman of the Board

Recent studies found that female CEOs or Chairman of the Boards are more risk-averse than their male counterparts. They are more likely to finance and invest more conservatively (Huang and Kisgen, 2013; Faccio et al., 2016), more conservative in accounting policies (Francis et al., 2015; Zalata et al., 2019), more likely to follow rules and regulations (Lanis et al., 2017; Adhikari et al., 2019), and take more responsibility in social events (McGuinness et al., 2017; Grosser and Moon, 2019). These researches raise concerns that our earlier findings may cause by the risk-aversion of female CEOs/chairmen and their tendency to hire female CFOs, not the risk-aversion of female CFOs themselves.

To alleviate the above concerns, we divided the full sample into female CEO vs. male CEO and female chairs vs. male chairs and tested the relationship between female CFOs and information disclosure violations, respectively. The results are in panel D of Table 5.

In the four subsamples of female CEO, male CEO, female chairs, and male chairs, the regression results represent that the coefficients on *Gender* are negative and significant at 5% level for our four measures of violations, indicating that our base results are not caused by the gender of CEOs/Chairman of the Board.

#### Different Sample Periods

As mentioned earlier, due to the time lag between the incidence of violation and revelation of regulators, we used 2016 as the end of our research period instead of 2018. It could be possible that the violation in 2016 may not be fully discovered; for this reason, we replaced our ending period with 2014 and 2015, respectively. The coefficients on *Gender* in Regressions (1) to (4) are negative and significant at 1–5% levels, as shown in panel E of Table 5.

#### Exogenous Shock

In addition, in 2007, the CSRC released the “Rule of information disclosure for Listed Companies,” which demonstrates the need of information disclosure and limits the CFO's violations. Hence, we took this event as an exogenous shock and divided the sample into 2003–2006 and 2007–2016, to see if female CFOs have a different impact on information disclosure violations. The results in panel F of Table 5 are similar to Table 2, representing that female CFOs can reduce firms' information disclosure violations before and after the regulation.

## Summary

Using China's listed companies from 2003 to 2016, we investigated whether female CFOs can reduce information disclosure violations. We found that female CFOs can reduce a firm's information disclosure violation because they have higher risk aversion and ethical moral standards.

To explore the moderate effect of CFO power on the relationship between CFO gender and disclosure violation, we followed Finkelstein (1992) and measured CFO power on four dimensions, namely, structural power, ownership power, expert power, and prestige power. The results showed that CFO power can significantly strengthen the effectiveness of female CFO in lowering the incidence of disclosure violation, and firms with more powerful female CFOs appear to have fewer violations. We finally studied the external monitoring from institutional investors, auditors, analysts, media, and local market, and the results represent that the negative relationship between female CFOs and violations is more pronounced for firms with stronger external monitoring.

Our results are robust after using different proxies in measuring violation, conducting a one-to-one PSM matching, controlling for the fact that the gender of CFO is an endogenous choice by using a Heckman self-selection model, partially controlling endogeneity with event study and DID method, ruling out the effect of female CEO/chairman, and employing different sample periods and exogenous shock.

## Data Availability Statement

The raw data supporting the conclusions of this article will be made available by the authors, without undue reservation.

## Author Contributions

YZ: main idea design and paper writing. JX and NY: data gathering and analyzing. JW: revision of the paper and reading proof. All authors contributed to the article and approved the submitted version.

## Funding

This study was funded by the National Natural Science Foundation of China (No. 71802101) and Shanghai Pujiang Program (No. 21PJC058).

## Conflict of Interest

The authors declare that the research was conducted in the absence of any commercial or financial relationships that could be construed as a potential conflict of interest.

## Publisher's Note

All claims expressed in this article are solely those of the authors and do not necessarily represent those of their affiliated organizations, or those of the publisher, the editors and the reviewers. Any product that may be evaluated in this article, or claim that may be made by its manufacturer, is not guaranteed or endorsed by the publisher.

## Footnotes

^1^The MI is a widely used index that measures the market and legal development in China's 31 provinces, autonomous regions, and municipalities from 1997 to 2014 (Fan et al., 2013; Lu et al., 2015). The index is jointly published annually by the National Economic Research Institute and the China Reform Foundation. Since our sample period extends beyond 2014, we matched cases after 2014 with the index value for 2014. Our results are qualitatively unchanged if we use the average index value between 2003 and 2014 instead.

^2^The Social trust index (Wang et al., 2017) is widely used in recent studies (Dong et al., 2018) and is collected from the 2017 report of the Business Environment Index for China's Provinces, which provides the magnitudes of social trust in 2006, 2008, 2010, 2012, 2014, and 2016 among China's provinces. Since the survey is conducted biannually, we do not have an index in 2007, 2009, 2011, 2013, and 2015. Following Dong et al. (2018), we used the index from the last year as the index for the current year. Our results are qualitatively unchanged if we use interpolation to fill in the data. For example, for 2007, we used the average indexes of 2006 and 2008.
