# Supplementary material for: CFO Gender, Corporate Risk-Taking, and Information Disclosure Violations
Source: Front Psychol. 2022 Jul 13;13:902472. doi: 10.3389/fpsyg.2022.902472 (PMC9326254; doi:10.3389/fpsyg.2022.902472)
Supplement: Supplementary file 1 [file Table_1.pdf]

Appendix A Variable definitions

Variable	Description
**Dependent Variables**	
Violation	An indicator variable equals to 1 if the firm has information disclosure violation in year t, and 0 otherwise.
Number	Total number of information disclosure violations which firm has in year t.
False	An indicator variable equals to 1 if the firm has false information disclosure violation in year t, and 0 otherwise. False violation means that CFO discloses false or misleading statements about a company (including false or misleading CSRC reports or financial statements).
Improper	An indicator variable equals to 1 if the firm has improper information disclosure violation in year t, and 0 otherwise. Improper violation relates to not disclose information on time, or disclose information which involve material omission or use accounting discretion improperly.
**Independent variable**	
Gender	An indicator variable equals to 1 if the CFO is female and 0 otherwise.
**Control variables**	
Size	Natural log of the total book value of assets
Lev	Long-term liabilities divided by total assets
ROE	Total income before extraordinary items divided by total assets
Loss	An indicator variable equals to 1 if the firm’s net income is below 0, and 0 otherwise.
Tobin’ Q	Market value of equity plus book value of total liability, then divided by total assets
SOE	An indicator variable equals to 1 if the firm is ultimately controlled by the government, and 0 otherwise.
List	The natural log of list year of the firm.
Big4	An indicator variable equals to 1 if the firm’s financial report is audited by the big 4 audit firms and 0 otherwise.
Board	The number of the board directors
Indep	Number of independent directors divided by the board size
Duality	An indicator variable equals to 1 if the COB and CEO are the same person, and 0 otherwise.
Age	Natural log of the age of CFO
Tenure	Natural log of the monthly tenure of CFO
Degree	An indicator variable equals to 1 if the CFO has a master’s of doctoral degree, and 0 otherwise.
Major	An indicator variable equals to 1 if the CFO’s major is accounting or finance in college, and 0 otherwise.
Abroad	An indicator variable equals to 1 if the CFO has studied or worked abroad before and 0 otherwise.
PC	An indicator variable equals to 1 if the CFO has past or concurrent work experience in the government or a political appointment, for example, a People’s Representative or a Member of Chinese People’s Political Consultative Conference is accounting or finance. Otherwise it is 0.
Year	Year dummies
Industry	Industry dummies
**Further analysis**	
Post	An indicator variable equals to 1 if the year is after the transition of a CFO, and 0 otherwise.
Up	An indicator variable equals to 1 if the transition if from a male CFO to a female CFO, and 0 otherwise.
Down	An indicator variable equals to 1 if the transition if from a female CFO to a male CFO, and 0 otherwise.
Power	An indicator variable equals to 1 if the CFO has high power and 0 if she has low power. The personal power of a CFO is measured by *Direct, Dcomp, Dshare, Dtenure, Dboard,* and *Dedu*. If one of the above measures equals 1, *Power* is also 1. If the 6 measure are 0, *Power* equals 0.

Direct	An indicator variable equals to 1 if the CFO is also the director of the board, and 0 otherwise.
Dcomp	An indicator variable equals to 1 if the CFO's total compensation (including salary, bonus, stock grants, and stock options) is higher than the industry-year median, and 0 otherwise.
Dshare	An indicator variable equals to 1 if the CFO holds the outstanding share of the firm, and 0 otherwise.
Dtenure	An indicator variable equals to 1 if the tenure of CFO is longer than the industry-year median, and 0 otherwise.
Dboard	An indicator variable equals to 1 if the CFO holds directorates on other corporate boards, and 0 otherwise.
Dedu	An indicator variable equals to 1 if the CFO holds a master's or doctoral degree, and 0 otherwise.
Gover	An indicator variable equals to 1 if the firm has high external monitoring and 0 if it has low monitoring. The external monitoring of a firm is measured by *Dinst, Daudit, Danalyst, Dmedia, DMI,* and *Dtrust*. If one of the above measures equals 1, *Gover* is also 1. If the 6 measure are 0, *Gover* equals 0.
Dinst	An indicator variable equals to 1 if the institutional investors' shareholding is greater than the industry-year median, and 0 otherwise.
Daudit,	An indicator variable equals to 1 if the financial report is audited by the big 4 auditor firms, and 0 otherwise.
Danalyst	An indicator variable equals to 1 if the number of analysts who follow the firm is greater than the industry-year median, and 0 otherwise.
Dmedia	An indicator variable equals to 1 if the firm's media coverage is greater than the industry-year median, and 0 otherwise.
DMI	An indicator variable equals to 1 if the marketization index (*MI*) of the province where firm locate is greater than the province-year median, and 0 otherwise.
Dtrust	An indicator variable equals to 1 if social trust index of the province where firm locate is greater than the province-year median, and 0 otherwise.

Appendix B Industry-year distribution of the sample

industry	2003	2004	2005	2006	2007	2008	2009	2010	2011	2012	2013	2014	2015	2016	Total
Agriculture, hunting, forestry, fishing	18	20	21	23	26	26	26	33	33	38	39	39	38	38	418
Mining	12	14	15	14	17	28	28	30	40	46	52	47	49	48	440
Manufacturing	474	550	599	603	618	667	684	765	982	1138	1231	1211	1245	1355	12122
Production and supply of electricity, heat, gas and water	34	37	40	44	47	48	51	51	57	60	58	56	56	56	695
Construction	15	18	17	20	27	30	31	30	33	42	46	43	44	48	444
Transportation, warehousing and postal services	36	38	43	39	46	45	44	42	48	58	59	61	60	64	683
Information transmission, software and information technology services	49	55	66	70	75	73	78	95	114	148	172	175	178	213	1,561
Wholesale and retail	58	61	72	73	80	82	82	101	107	124	125	117	121	129	1,332
Real estate	40	39	44	43	47	59	70	81	91	92	96	98	103	92	995
Social Services	29	28	28	31	34	34	33	44	50	54	64	61	67	71	628
Communication and Cultural Industries	9	9	8	6	7	8	9	7	19	25	33	30	33	37	240
Comprehensive	60	70	62	53	57	49	49	56	39	40	40	41	38	46	700
Total	834	939	1,015	1,019	1,081	1,149	1,185	1,335	1,613	1,865	2,015	1,979	2,032	2,197	20,258

Industries are classified according to the code of China Securities Regulatory Commission (CSRC), edition 2001.
